# Disease-Modifying Adjunctive Therapy (DMAT) in Osteoarthritis—The Biological Effects of a Multi-Mineral Complex, LithoLexal^®^ Joint—A Review

**DOI:** 10.3390/clinpract11040104

**Published:** 2021-11-26

**Authors:** Erik Fink Eriksen, Osvandre Lech, Gilberto Yoshinobu Nakama, Denise M. O’Gorman

**Affiliations:** 1 Spesialistsenteret Pilestredet Park and Department of Odontology, University of Oslo, 0315 Oslo, Norway; 2 Shoulder and Elbow Service, Hospital Sao Vicente de Paulo, and Instituto de Ortopedia e Traumatologia, R. Uruguai, 2050-Centro, Passo Fundo 99010-112, Brazil; lech.med@terra.com.br; 3 Articulare Clínica Integrada, Alameda dos Maracatins, 1217-Cj. 517-Indianópolis, São Paulo 04089-014, Brazil; gilbertoyn@gmail.com; 4 Marigot Ltd., Carrigaline Co., P43 E409 Cork, Ireland; Denise.ogorman@marigot.ie

**Keywords:** osteoarthritis, LithoLexal, lithothamnion, disease-modifying adjunctive therapy, anti-inflammatory agents, cytokine inhibitors

## Abstract

Modern advances in molecular medicine have led to the reframing of osteoarthritis as a metabolically active, inflammatory disorder with local and systemic contributing factors. According to the ‘inflammatory theory’ of osteoarthritis, immune response to an initial damage is the key trigger that leads to progressive joint destruction. Several intertwined pathways are known to induce and govern articular inflammation, cartilage matrix degradation, and subchondral bone changes. Effective treatments capable of halting or delaying the progression of osteoarthritis remain elusive. As a result, supplements such as glucosamine and chondroitin sulphate are commonly used despite the lack of scientific consensus. A novel option for adjunctive therapy of osteoarthritis is LithoLexal^®^ Joint, a marine-derived, mineral-rich extract, that exhibited significant efficacy in clinical trials. LithoLexal^®^ has a lattice microstructure containing a combination of bioactive rare minerals. Mechanistic research suggests that this novel treatment possesses various potential disease-modifying properties, such as suppression of nuclear factor kappa-B, interleukin 1β, tumor necrosis factor α, and cyclooxygenase-2. Accordingly, LithoLexal^®^ Joint can be considered a disease-modifying adjunctive therapy (DMAT). LithoLexal^®^ Joint monotherapy in patients with knee osteoarthritis has significantly improved symptoms and walking ability with higher efficacy than glucosamine. Preliminary evidence also suggests that LithoLexal^®^ Joint may allow clinicians to reduce the dose of nonsteroidal anti-inflammatory drugs in osteoarthritic patients by up to 50%. In conclusion, the multi-mineral complex, LithoLexal^®^ Joint, appears to be a promising candidate for DMAT of osteoarthritis, which may narrow the existing gap in clinical practice.

## 1. Introduction

Osteoarthritis is a degenerative joint disease characterized by articular cartilage erosion, subchondral bone pathology and sclerosis, marginal bone hypertrophy and a range of biochemical and morphological alterations of the synovial membrane and joint capsule. Although it can affect any joint, osteoarthritis occurs most often in knees, hips, lower back, neck, small joints of the fingers, base of the thumb and big toes. Clinical diagnosis relies on patient’s history, physical examination, and radiographic evidence [1]. Medical management of osteoarthritis still poses a significant clinical challenge, and its outcomes, in most cases, are far from satisfactory. Considering this, the current review aims to introduce the potential position and clinical efficacy of an emerging osteoarthritis adjunctive therapy (LithoLexal^®^ Joint, Nordic Medical Ltd., London, UK) and elaborate on our current state of understanding of its mechanisms and action.

### 1.1. Epidemiology and Health Burden of Osteoarthritis

Osteoarthritis is the most common chronic condition of the joints and the single most common cause of disability in older adults. Ageing is the main risk factor for developing osteoarthritis given that its prevalence considerably increases with age. For instance, the prevalence of knee osteoarthritis increases from 26% in persons younger than 60 years to 44% in individuals older than 70 years [2]. Obesity is another major risk factor, strongly associated with knee and hand osteoarthritis so that every 5-unit increase in body mass index elevates the risk of knee osteoarthritis by 35% [3]. This chronic disease more often occurs in women than men and in developed than developing countries [4]. Evidently, radiographic hand osteoarthritis is the most prevalent form that occurs in 27–80% of adults, while depending on the population, hip osteoarthritis has a prevalence rate of up to 45% [5].

Advanced osteoarthritis can lead to significant psychological, social and economic consequences and impose a substantial financial burden to the health system [6]. Due to ageing of the population and a growing obesity pandemic, the magnitude of this burden is increasing. According to the 2010 Global Burden of Disease Study, hip and knee osteoarthritis ranked 11th highest in terms of ‘years of life lived with disability’ among 291 conditions evaluated [7]. It is estimated that this chronic joint disease is responsible for ~7% of the ‘total productive life years’ lost due to disability worldwide [8].

### 1.2. Understanding Osteoarthritis as an Inflammatory Disease 

**Figure 1:**
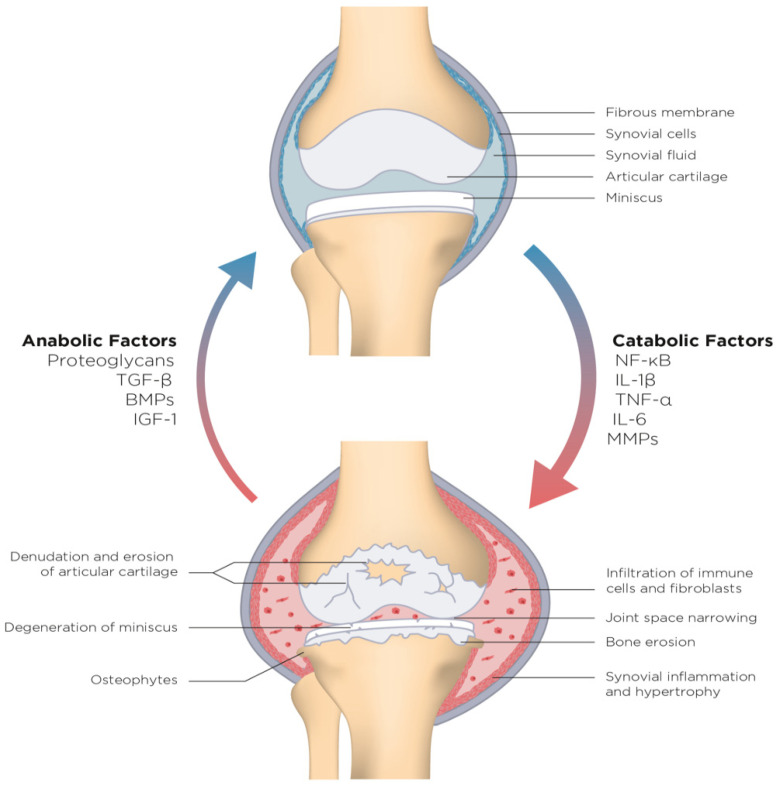
In osteoarthritic joints, a stimulated increase in the production of proinflammatory cytokines causes an overactivity of catabolic factors that provoke degenerative changes to all articular tissues. In response, anabolic pathways set out in vain to increase matrix production and repair the tissue damage. Exaggerated responses may lead to hypertrophy of chondrocytes and osteophyte formation.

Contemporary advances in molecular medicine have increased our understanding of the pathophysiology of osteoarthritis. It is now well known that osteoarthritis is a multifactorial, metabolically active condition that involves all joint tissues, i.e., articular cartilage, synovium, subchondral bone, menisci, ligaments, muscles and nerves [9]. In this model, low-grade, chronic inflammation plays a central role as the mediating mechanism that connects etiological factors to a pervasive tissue damage characteristic of advanced arthritis [10]. Direct evidence has recently become available through RNA sequencing that a large number of the signaling pathways in osteoarthritic patients are associated with immune response and inflammation [11,12]. To reflect this novel development, the phrase ‘tear, flare and repair’ has been proposed [13], signifying the fundamental role of immune response in the pathogenesis of osteoarthritis. The term ‘tear’ represents focal biomechanical insults resulting from overload, overuse, malalignment, or injury, which lead to fibrillation, erosion, and cracking on the superficial layer of articular cartilage. Damage-associated molecules released into the synovial fluid during this initial tear can induce inflammatory reactions in synovial macrophages and catabolic responses in chondrocytes [14], referred to as ‘the flare’. In the clinic, synovial inflammation and thickening can be observed as morning stiffness, joint warmth, and effusion. Irritated synovial cells produce inflammatory cytokines, including interleukin 1β (IL-1β), IL-6 and tumor necrosis factor α (TNF-α), that share a capacity to activate an array of catabolic genes and signaling pathways in chondrocytes. Inflammatory signals stimulate cytokine-activated transcription factors, such as nuclear factor kappa-B (NF-κB), and thereby inhibit the activity of collagen promoters and decrease the production of cartilage extracellular matrix [15] (Figure 1). For instance, IL-1β inhibits the production of aggrecan [16] and types II and IX collagen fibers [17]. 

Another significant process contributing to pain and disability in osteoarthritis is the formation of bone marrow lesions in the subchondral bone [18]. This phenomenon is considered to be a repair process in response to persistent trauma, which contributes to excessive levels of cytokines and angiogenetic factors and causes increasing pain due to modulation of nociceptive pathways [19]. In addition, the generation of oxidative and nitrosative acting substances is triggered by inflammatory processes. In the presence of depleted antioxidants in affected cartilages, oxidative stress further contributes to impaired biological activities, cell death and breakdown of matrix components [20].

Another impact of proinflammatory cytokines that disturbs the balance of cartilage remodeling processes is promoting the expression of matrix metalloproteinases (MMPs) by both chondrocytes and synovial cells [21]. The discovery of this direct association between soluble inflammatory mediators and MMPs was a major milestone in understanding the pathogenesis of cartilage loss in osteoarthritis. It is currently well documented that upregulated or inappropriately activated MMPs, especially MMP-13 and aggrecanases, are responsible for proteoglycan and collagen degradation under the conditions of osteoarthritis [22]. In parallel, the provoked metabolic stress in chondrocytes can result in the loss of viable cells owing to apoptosis or senescence [23]. 

Reacting to partial-thickness injuries, in a specific reparation process called ‘intrinsic repair’, chondrocytes of the hyaline cartilage tissue initially proliferate and synthesize an enhanced quantity of proteoglycans and collagen fibers. However, the repair tissue usually has neither the original structure nor the properties of a normal cartilage and is more susceptible to decay. Even this inept response will soon be outmatched by the ongoing insults and protease activity that shift the cartilage remodeling balance in favor of net degradation and loss of cartilage matrix [24]. In summary, the development of chronic inflammation and tissue destruction in osteoarthritis can be understood as a self-perpetuating cycle of local tissue damage, inflammation and ineffective repair in such a way that the osteoarthritic joint has been likened to a chronic wound [25].

## 2. The Role of Adjunctive Therapy in the Management of Osteoarthritis

The ultimate goal in clinical treatment of osteoarthritis is to modify its pathogenetic processes and thereby prevent or delay permanent joint damage, which leads to serious disability. To this end, a comprehensive management plan is employed comprising of nonpharmacologic conservative modalities, topical and oral analgesics, e.g., nonsteroidal anti-inflammatory drugs (NSAIDs), and intraarticular corticosteroids together with different forms of adjunctive therapy to address the multi-factorial nature of the disorder. Conservative interventions for the treatment of pain in osteoarthritis include physical therapy and exercise as well as low-level laser therapy. These conventional modalities frequently fail to provide significant improvement in pain at short- and long-term, thus their sufficiency and clinical merit have been questioned by some authors [26]. In this context, a timely utilization of effective adjunctive therapy is essential, knowing that most first-line treatments are palliative and lack disease-modifying efficacy. Besides, a delayed intervention in the advanced stages of osteoarthritis with potent medications, as per current protocols, rarely results in altering the malign course of the disease.

### 2.1. Conventional Adjunctive Therapies

A range of products containing different forms of glucosamine (GluN) and chondroitin sulphate (CS) have been in clinical use for years. However, the evidence for their efficacy has always been controversial. In the largest randomized controlled trial on the subject to date, the Glucosamine/chondroitin Arthritis Trial (GAIT) [27], the response rate in knee osteoarthritic patients with either CS or its combination with GluN was relatively small and not significantly different from placebo after 24 weeks. Only a modest symptomatic response was observed with GluN + CS in patients with moderate-to-severe pain. Later, a meta-analysis of the results from 10 large controlled trials has concluded that based on the magnitude of change in outcome measures, GluN and/or CS were not clinically different from placebo in improving joint pain or joint space width in knee or hip osteoarthritis [28]. With regard to the hip, monotherapy with GluN failed to reduce the symptoms and progression of osteoarthritis after two years [29]. Based on the above, health authorities gave both CS and GluN uncertain recommendations for symptom relief and emphasized that these supplements are not appropriate for disease modification [30].

### 2.2. Natural Multi-Mineral Complexes

**Figure 2:**
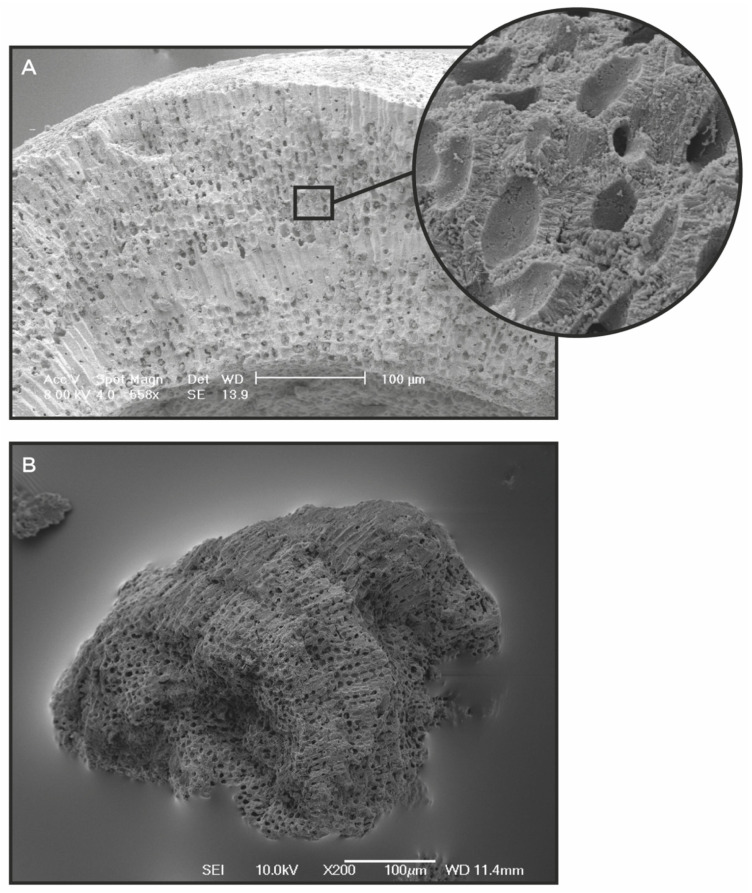
(**A**) The microscopic structure of mineralized skeleton of Lithothamnion as revealed by scanning electron microscope; (**B**) The structure of a LithoLexal^®^ particle after being extracted and milled indicating a similar lattice structure to the source; Images are courtesy of Marigot Ltd. (West Sussex, UK).

More than two decades ago, a new form of osteoarthritis adjunctive therapy had been introduced. This novel treatment (LithoLexal^®^ Joint, Nordic Medical Ltd., London, UK) is based on multi-mineral complexes extracted from Lithothamnion species, a genus of red algae that produces calcareous skeleton. Bio-mineralization by this marine organism is exerted via condensing and precipitating biologically active sea minerals in its cell walls and extracellular spaces through both biologically induced and biologically controlled mineralization processes [31]. Imaging and analytical techniques have confirmed that Lithothamnion biominerals are principally arranged as high-magnesium calcite, aragonite, dolomite, and magnesite microstructures [32]. This unique intricate structure (Figure 2A) has been harvested for human use through a proprietary extraction technique to preserve the original structure of the source material (Figure 2B). The final product has a spongy microstructure containing more than 70 macro and trace biominerals [33]. This multi-mineral extract, previously produced under the brand name of Aquamin^®^, is now solely licensed to and is produced by Nordic Medical LTD (London, UK) marketed as LithoLexal^®^. A porous and lattice structure affords LithoLexal^®^ a high surface to mass ratio and improved solubility profile compared to rock-based products. 

LithoLexal^®^ complex has been proved bioactive in treating several health conditions with inflammation as an underlying mechanism including postmenopausal osteoporosis [34,35], colitis [36], gastrointestinal polyposis [37] and ulcerative dermatitis [38]. This putative anti-inflammatory property and benign safety profile have made LithoLexal^®^ a promising option for adjunctive therapy of osteoarthritis. In later sections, evidence for efficacy and the position of this novel treatment in a comprehensive osteoarthritis management regimen are described. 

### 2.3. Oral Proteoglycan Replacement Therapy (PRT)

Therapeutic effects of oral administration of specific marine-derived proteoglycans have been evaluated in a variety of inflammatory conditions, including allergic respiratory inflammation [39], experimental colitis [40] and alopecia [41] with promising outcomes. In-vivo studies have unveiled some aspects of the anti-inflammatory properties of this novel treatment. Observations indicated that oral administration of marine-derived proteoglycans can downregulate interferon-β and γ in addition to multiple proinflammatory interleukins [42]. 

In addition to anti-inflammatory attributes, the ability of PRT to enhance the concentration of cartilage proteoglycans is especially relevant in the therapeutic approach towards osteoarthritis. Cartilaginous tissues are rich in proteoglycans among which aggrecan is the most abundant and functionally important proteoglycan, which provides resistance against compressive loads. Both of its core protein and polysaccharide chains are susceptible to degradation by enzymes and reactive oxygen species [43], a phenomenon that evidently occurs in osteoarthritis [44]. Accumulating evidence suggests that the augmented presence of cytokines, especially IL-1 and TNF-α, in osteoarthritic joints disturbs the turnover of cartilage proteoglycans by both suppressing their synthesis and upregulating the proteases [45]. A reduced concentration of proteoglycans leads to disturbed fluid pressurization within the cartilage and loss of tensile support for collagen fibrils and thus permanent damage and loosening of the collagen network [43]. Since this phenomenon usually happens early in the course of osteoarthritis, it is believed to play a causal role.

Due to the fact that proteoglycans are actively synthesized and deposited into the cartilage throughout a person’s lifetime and their early-stage depletion is ‘reversible’ [21], enhancing the production of proteoglycans in osteoarthritis-affected cartilages is a viable intervention target. To meet this target, a specific blend of fish cartilage proteoglycans, branded as Vercilexal^®^ (Nordic Medical Ltd., London, UK), has been developed as a form of joint PRT. Preliminary clinical research has confirmed the mitigating effect of long-term oral treatment with marine-derived proteoglycans on the symptoms of knee osteoarthritis [46] and knee discomfort [47]. Evaluation of cartilage metabolism indicated that marine-derived proteoglycans significantly diminished collagen degradation and promoted collagen synthesis in subjects with severe pain [47]. However, recognizing the full potential and action mechanism of joint PRT with Vercilexal^®^ in different types of osteoarthritis demands more mechanistic as well as clinical investigations.

## 3. Disease-Modifying Adjunctive Therapy (DMAT) in Osteoarthritis: A Novel Frontier

A disease-modifying drug by definition is a pharmaceutical that targets one (or more) of the underlying pathologic process(es) of a disease whereby it reduces the severity and/or frequency of symptoms and delays or prevents disease progression and complications. This concept has first been promulgated for a class of antirheumatic drugs but has later been adopted by other fields of medicine, particularly neurology, to describe other families of drugs. In case of dietary supplements and adjunctive therapies, a mounting body of evidence has become available during the past few decades denoting that some natural compounds are able to modify the course of certain diseases, a good example being alpha linolenic acid in stroke [48]. Therefore, the term ‘disease-modifying adjunctive therapy (DMAT)’ has been suggested to differentiate the compounds with disease-modifying efficacy from symptom-modifying agents. 

With regard to osteoarthritis, no conventional adjunctive therapy is proven effective in modifying the progression of joint damage, and thus a clinical need for an osteoarthritis DMAT still exists. Based on the modern ‘inflammatory theory’ of osteoarthritis, it is a prerequisite for a compound to demonstrate efficient anti-inflammatory properties before it can qualify as an osteoarthritis DMAT. Given this, the natural multi-mineral complex, LithoLexal^®^, has been proposed as a potential candidate on grounds of its anti-inflammatory properties reported by several lines of mechanistic research. Through a controlled in-vitro experiment [49], scientists have uncovered that pre-treatment of macrophages with LithoLexal^®^ can significantly reduce the level of lipopolysaccharide-induced NF-κB transcriptional activity compared with untreated cells. Suppression of NF-κB by LithoLexal^®^ was a result of enhancing the activity of its inhibitory protein, the inhibitor of kappa B alpha (I-κBα). NF-κB protein complex is upregulated by TNF-α, IL-1β, reactive oxygen species and other stress-related factors. Hence, similar to other inflammatory diseases, NF-κB dysregulation is implicated in the pathophysiology of osteoarthritis. Accordingly, targeted strategies that interfere with NF-κB signaling could offer unconventional therapeutic options for osteoarthritis [50].

LithoLexal^®^ also demonstrates a considerable inhibitory effect on cyclooxygenase-2 (COX-2) expression. In a study by O’Gorman et al. [49], COX-2 expression in lipopolysaccharides (LPS)-stimulated macrophages treated with either LithoLexal^®^ or placebo was compared. Observations corroborated that a physiologically relevant concentration of LithoLexal^®^ is effective in diminishing the cellular expression of COX-2. Prostaglandins, particularly prostaglandin E2, are among the mediators that not only stimulate the production of cartilage-degrading proteases, but also contribute to osteoarthritis-associated pain pathways and hyperalgesia. Prostaglandin E2 sensitizes peripheral nociceptors via cAMP-mediated enhancement of sodium currents [51]. Since IL-1β is a potent inducer of COX-2, the inhibition of IL-1β by LithoLexal^®^, described earlier [52], may represent a synergistic, analgesic activity that further augments the impact of LithoLexal^®^ on prostaglandin suppression. This reflects a key therapeutic effect for the fact that pain is the most prominent and disabling symptom of osteoarthritis and thus is a main target of medical treatment.

Suppressing the secretion of proinflammatory cytokines can theoretically dissociate the initial biomechanical insult from its consequent intraarticular and systemic inflammation in osteoarthritic subjects. From this perspective, the potent ability of physiologic concentrations of LithoLexal^®^ to attenuate the induced secretion of both TNF-α and IL-1β by macrophages [52] suggests a disease-modifying potential. The initial findings were subsequently verified by a controlled double-blinded human trial in which monotherapy by LithoLexal^®^ decreased the serum levels of TNF-α in individuals with moderate to severe osteoarthritis [53]. This is important since previous research has associated the elevated serum levels of TNF-α with severity of osteoarthritis and its future progression [54]. 

Based on the above, marine-derived multi-mineral complexes can be taken as promising therapeutics with several potential disease-modifying effects for the management of osteoarthritis. Considering that inflammatory effectors act in a parallel redundant manner in osteoarthritis [10], the multi-dimensional bioactivity of LithoLexal^®^ may help in controlling the progression of this debilitating disorder.

## 4. LithoLexal^®^ Joint, a Natural DMAT for the Management of Osteoarthritis

The anti-inflammatory properties of LithoLexal^®^ Joint underpinning its application as a DMAT for the prevention of joint damage were described earlier. In this section, clinical trials of monotherapy and adjunctive therapy with LithoLexal^®^ Joint in individuals with mild to severe osteoarthritis will be reviewed.

In a randomized, active-controlled, clinical trial, LithoLexal^®^ was compared with placebo, GluN alone and a combination of GluN plus LithoLexal^®^ in men and women with knee osteoarthritis [55]. After 12 weeks of intervention, monotherapy with LithoLexal^®^ significantly improved pain, stiffness, activity and composite scores of the Western Ontario and McMaster Universities (WOMAC) index compared to placebo and produced larger effect sizes than GluN. The walking capacity of patients, evaluated by six-minute walk test, was improved by both supplements; however, the increase in walking distance with LithoLexal was almost twice that of GluN. An unexpected but important conclusion of this study was that the combination of LithoLexal^®^ and GluN failed to induce any significant clinical improvements probably due to an antagonistic pharmacological effect [55]. A subsequent placebo-controlled trial [56] set out to investigate the potential of LithoLexal^®^ adjunctive therapy to allow for reducing the dosage of NSAIDs in advanced osteoarthritic patients. Researchers tapered the usual daily dosage of NSAIDs by 50% after two weeks of coadministration with LithoLexal^®^, and then tapered again after four weeks to discontinuation. One month into the study, individuals on LithoLexal^®^ exhibited a dramatically improved six-minute walking distance while only receiving half of their usual dose of NSAIDs. The tapered need of patients for NSAIDs may root in the COX-2 suppressive activity of LithoLexal^®^ described earlier. It is also likely that mineral supplementation via LithoLexal^®^ Joint imparts a palliative influence on myofascial pain syndrome due to central sensitization that frequently exists in patients with chronic painful osteoarthritis. Research revealed that the majority of patients with knee osteoarthritis have an increased number of active and latent myofascial trigger points, which are a source of painful motion [12,57]. Certain trace minerals, such as magnesium, selenium and zinc, are promising options for the treatment of central sensitization syndromes as elaborated in a review by Aguilar-Aguilar et al. [58].

As with walking capacity, active and passive range of motion in extension have also significantly improved by LithoLexal^®^, whereas between-group differences in WOMAC index and rescue medication consumption did not reach statistical significance. Importantly, dropouts due to aggravated pain after NSAID discontinuation were five times lower in LithoLexal^®^ than in the placebo group, indirectly indicating a higher symptom-relieving efficacy of LithoLexal^®^ [56]. Recently, Heffernan et al. a crossover, active-controlled trial that confirms the pain-relieving effect of LithoLexal^®^ in osteoarthritic patients. In this study, the efficacy of 12 weeks of treatment with LithoLexal^®^ (plus seawater derived MgOH_2_ and pine bark extract) was compared with GluN. At endpoint, only LithoLexal^®^ improved pain in both affected men and women (*p* < 0.01). Similar to the study by Frestedt et al., LithoLexal^®^ caused a meaningful decline of 72% (*p* = 0.03) in patients’ overall need for analgesics, 35% of which were NSAIDs. The functional performance of subjects was also improved by LithoLexal^®^ as measured by ‘timed-up-and-go’ test. Treatment safety and tolerability were excellent reflected in a 93% treatment adherence throughout the study [59]. An independent trial was also carried out by Murphy et al., to assess how LithoLexal^®^ affects inflammatory biomarkers and typical symptoms of osteoarthritis. Seven out of 11 participants had low WOMAC composite index score after six weeks of LithoLexal^®^ therapy; specifically, eight felt less pain, six felt less stiffness and six felt an improvement in performing their daily activities. Additionally, the key inflammatory biomarker, TNF-α, was reduced by 23.6% compared to the baseline. Adding antioxidant bioflavonoids intensified the efficacy of LithoLexal^®^ in this setting [53]. It has to be considered that, due to a short intervention period, this trial is likely to have underestimated the effect size of LithoLexal^®^ Joint. Equally important, no clinically significant side effects or dropouts related to LithoLexal^®^ Joint therapy has been observed during its clinical testing.

As yet, the exact action mechanism of this marine-derived multi-mineral extract in modulating the pathogenesis of osteoarthritis is not fully understood and demands further research. Nevertheless, it can be conjectured that the ability of LithoLexal^®^ in suppressing the inflammatory response of macrophages via downregulating several key proinflammatory pathways [49,52,53] plays a sizable role in its clinical bioactivity. Moreover, there are clues in the literature that some biominerals included in the structure of LithoLexal^®^ possess in-vivo anti-inflammatory and antioxidative properties and can support cartilage formation, interfering with the progression of osteoarthritis. According to recent observations, high intake of magnesium is inversely associated with radiographic knee osteoarthritis and joint space narrowing [60]. This finding may be explained by the enhancing effects of magnesium on the synthesis of cartilage matrix and promotion of the adhesion of human synovial stem cells through integrins [61]. On the other hand, lower magnesium intake is associated with more severe pain and reduced function in subjects with knee osteoarthritis [62]. This association is conceivably due to increased expression of TNF-α and resulting activation of NF-kB. Beyond this, magnesium has been speculated to have analgesic effects [63]. Manganese is another trace mineral with beneficial potentials for joint health. This biomineral is the preferred cofactor of glycosyltransferases required for the synthesis of proteoglycans during cartilage formation. Manganese also takes part in the function of a member of superoxide dismutase family of antioxidant enzymes mainly in mitochondrial matrix. This enzyme limits the formation of extremely reactive oxygen species, such as hydroxyl radical and peroxy-nitrite, that cause cartilage degradation by activating MMPs [64]. Gene expression studies have shown that the level of manganese superoxide dismutase is reduced in osteoarthritis affected cartilages, which can be detected even prior to the appearance of cartilage erosions [65]. 

Modulation of gut microbiome is yet another plausible mechanism via which LithoLexal^®^ Joint mitigates local and systemic inflammation in osteoarthritic patients. This hypothesis originates from the novel concept of intestine–brain–articulation axis and its role in the association between low-grade inflammation and osteoarthritis. It is well-established that gut microbiota can influence the pathophysiology of several distant organs, including the skeletal system. In the case of osteoarthritis, the dysbiosis of intestinal microbiome may lead to elevated absorption of proinflammatory microbial products, such as LPS, which can activate macrophage cells in the knee and aggravate disease severity and joint symptoms, mainly pain [66,67]. A factor with promising potential to break this cycle by altering the diversity of gut microbiome is the use of dietary trace minerals. Of note is that the interaction between gut microbiome and minerals is bidirectional meaning that while the microbiota can affect the absorption of minerals, its composition is also influenced by changes in the level of trace minerals. For instance, selenium status strongly correlates with a higher diversity of gut microbiome [68]. Therefore, it is not unreasonable to propose that LithoLexal^®^ Joint as a source of major and trace biominerals may provoke observable improvements in the dysbiotic microbiome of patients with osteoarthritis.

Overall, a substantial synergy between major biominerals included in the unique composition of LithoLexal^®^ Joint may underlie its in vivo pharmacological functions and therapeutic effects on cartilage formation and maintenance in osteoarthritis affected joints.

## 5. Conclusions 

The contemporary evolution in our understanding of the causal role of proinflammatory pathways and cytokines in the pathogenesis of osteoarthritis has posed an opportunity for the development of disease-modifying drugs and supplements. As a result, several natural and synthetic compounds have been tested; however, a safe treatment with inflammation-suppressing properties has been elusive. The concept of DMAT in osteoarthritis evolves from this paradigm-shifting perspective with the introduction of a marine-sourced, multi-mineral complex, LithoLexal^®^. This specific bioactive complex has exhibited multi-dimensional, inflammation-suppressing activities on macrophage cell lines, which appears to be translated into clinical efficacy as shown by a number of clinical trials. Improvements in all subscales of WOMAC index and a significant increase in the ability of patients to walk after a relatively short treatment period with LithoLexal^®^ Joint are indicative of potent clinical efficacy. These promising findings, however, await further confirmation by larger studies.

Known to generate a multiplicity of major adverse effects, most disease-modifying medications are merely indicated for patients with advanced stages of osteoarthritis. To help individuals with milder forms of the disease, symptomatic slow-acting drugs for osteoarthritis (SySADOA) have been introduced over recent decades [69]. LithoLexal^®^ Joint can be considered a novel form of SySADOA and an effective option for primary or secondary prevention of clinical or radiological osteoarthritis with a desirable safety and tolerability profile. LithoLexal^®^ Joint can be administered as a mono- or adjunctive therapy to conventional osteoarthritis medications depending on the needs of each individual. Importantly, there is evidence to support that coadministration of LithoLexal^®^ Joint with NSAIDs can reduce the overall need for analgesia by half and hence mitigate their burden of side effects. Diverse disease-modifying properties of LithoLexal^®^ can conceivably modulate articular inflammation and prevent its self-aggravation and perpetuation.

In summary, LithoLexal^®^ Joint is a promising option for adjunctive therapy of patients with mild to severe osteoarthritis with putative multi-dimensional, disease-modifying effects, shown to improve patients’ symptoms and mobility and reduce the need for analgesics. Future research is expected to illuminate more details on the pharmacology and clinical application of LithoLexal^®^ Joint in the prevention and management of one of the most common chronic diseases faced by humans.

## Data Availability

All the data supporting the conclusions of this review are published in scientific journals.
